# Astaxanthin Protects Human Granulosa Cells against Oxidative Stress through Activation of NRF2/ARE Pathway and Its Downstream Phase II Enzymes 

**DOI:** 10.22074/cellj.2021.7222

**Published:** 2021-07-17

**Authors:** Mojtaba Eslami, Sahar Esfandyari, Marzieh Aghahosseini, Zahra Rashidi, Shirzad Hosseinishental, Samane Brenjian, Aligholi Sobhani, Fardin Amidi

**Affiliations:** 1. Department of Anatomy, School of Medicine, Tehran University of Medical Sciences, Tehran, Iran; 2. Department of Infertility, Shariati Hospital, Tehran University of Medical Sciences, Tehran, Iran; 3. Fertility and Infertility Research Center, Health Technology institute, Kermanshah University of Medical Sciences, Kermanshah, Iran

**Keywords:** Astaxanthin, Granulosa Cells, Nuclear Factor-E2-Related Factor 2, Oxidative Stress

## Abstract

**Objective:**

Astaxanthin (AST) has been introduced as a radical scavenger and an anti-apoptotic factor that acts via regulating the nuclear factor-E2-related factor 2 (NRF2) and related factors. Here, we intended to examine the effect of AST on granulosa cells (GCs) against oxidative stress by examining NRF2 and downstream phase II antioxidant enzymes.

**Materials and Methods:**

In this experimental study, we used cultured human primary GCs for the study. First, we performed the 3-(4,5-dimethylthiazol-2-yl)-2,5-diphenyltetrazolium bromide (MTT) test to evaluate cells viability after treatment with hydrogen peroxide (H2O2) and AST. The apoptosis rate and ROS levels were measured by flow cytometry. To determine NRF2 and phase II enzymes expression, we performed real-time polymerase chain reaction (PCR). Finally, we used western blot to measure the protein levels of NRF2 and Kelch-like ECsH-associated protein 1 (KEAP1). Enzyme activity analysis was also performed to detect NRF2 activity.

**Results:**

This study showed that AST suppressed ROS generation (P<0.01) and cell death (P<0.05) in GCs induced by oxidative stress. AST also elevated gene and protein expression and nuclear localization of NRF2 and had an inhibitory effect on the protein levels of KEAP1 (P<0.05). Furthermore, when we used trigonelline (Trig) as a known inhibitor of NRF2, it attenuated the protective effects of AST by decreasing NRF2 activity and gene expression of phase II enzymes (P<0.05).

**Conclusion:**

Our results presented the protective role of AST against oxidative stress in GCs which was mediated through up-regulating the phase II enzymes as a result of NRF2 activation. Our study may help in improving in vitro fertilization (IVF) outcomes and treatment of infertility.

## Introduction

Oxidative stress as a result of disruption in reduction-oxidation (redox) homeostasis is an unavoidable threat for different human cells. When a great amount of reactive oxygen species (ROS) is generated, cells become more sensitive to outcomes of oxidative stress including apoptosis and damages to major organic molecules. For a better understanding it should be noted that oxidative stress is a caused by as an imbalance between the production of ROS and antioxidant scavengers levels (1). In normal states, a complex antioxidant system with several important defense enzymes, protects cells against oxidative stress through scavenging ROS and maintaining the redox homeostasis. Among these endogenous scavengers, nuclear factor-E2-related factor 2 (NRF2)/ Kelch-like ECH-associated protein 1 (KEAP1)-antioxidant response element (ARE) pathway and its underlying mechanism involving phase II enzymes including glutamate-cysteine ligase (GCL), heme oxygenase 1 (HO1), and NAD(P)H quinone dehydrogenase 1 (NQO1), regulate antioxidant responses. GCL holoenzyme is an important antioxidant in glutathione biosynthesis with two different subunits, GCLC as a catalytic subunit and GCLM as a modifier subunit. HO1 cleaves the heme ring and leads to the formation of biliverdin and subsequently, bilirubin as potent antioxidants. Moreover, an excess amount of heme sensitizes cells to apoptosis. NQO1, as a flavoprotein can be produced under different stress conditions particularly oxidative stress in order to reduce quinones to hydroquinones and prevent the formation of subsequent ROS (2). Therefore, when ROS production is increased, NRF2 as a key transcription factor, translocates into the nucleus and enhances the expression of phase II antioxidant enzymes by attaching to ARE region but under normal conditions, KEAP1 binds to NRF2 and facilitates its degradation through ubiquitination. Any disturbance in the function of this pathway results in an inability to neutralize the oxidative stress and following damages to multiple cells (3).

It is widely known that oxidative stress is highly correlated with chronic inflammation, age-related diseases, cancer, and infertility in both men and women. Over the past years, considerable effort has been made to increase the success rate of infertility treatments. Oxidative stress is regarded as an imperative factor affecting the success rates of *in vitro* fertilization (IVF) especially in granulosa cells (GCs) of women with polycystic ovarian syndrome (PCOS) (4). GCs surround the oocyte within the developing ovarian follicles and are key cells for the production of steroids as well as growth factors required for ovarian follicles growth and function. Although maintenance of normal physiological levels of ROS is important for successful fertilization and regulation of spermatozoa maturation, capacitation, hyperactivation, acrosomal reaction, chemotactic processes, and sperm-oocyte fusion, the overproduction of ROS has been linked to many fertility complications caused by damaging many organic molecules. Since oxidative stress reverses well maturation of GCs and embryo quality, a great attempt must be made for management of ROS generation (5).

Astaxanthin (AST, 3,3′-dihydroxy-β,β′-carotene-4,4′- dione) is a powerful carotenoid pigment naturally found in orange and red fruits. AST with multiple health benefits has a wide range of biological activities including antioxidant, anti-apoptotic, anti-inflammatory, and neuroprotective effects. AST with a great antioxidant capacity can inhibit oxidative damage and then, protect different cells from most pathological conditions. Previous studies reported that AST shows significant antioxidant activities not only through radical scavenging but also by inducing the expression of NRF2 and its downstream target genes, to promote the antioxidant defense in human cells (6). However, there is no study indicating the protective role of AST in human GCs against oxidative stress. Since oxidative damage is a major cause of GCs and oocytes apoptosis and subsequent infertility in women, we intended to examine the possible role of AST in protecting cultured primary human GCs against hydrogen peroxide (H_2_ O_2_)-induced oxidative stress through up-regulation of NRF2 pathway and subsequent activation of phase II enzymes including GCL, HO1, and NQO1. Furthermore, trigonelline (Trig) was used as an inhibitor of NRF2 (7) to express the link between NRF2-ARE pathway and AST-induced phase II enzymes expression.

## Materials and Methods

This study was approved by the Ethics Committee of Tehran University of Medical Sciences (IR.TUMS. MEDICINE.REC.1395.730) and written informed consent was obtained from all contributors before initiation of the research.

### Study population and granulosa cells isolation and culture

Our study was an experimental study. GCs used in the current study were provided from follicular fluid of healthy women aged between 20-38 years old, who had a regular menstrual cycle and healthy ovulatory function, were not taking took no hormonal drugs and underwent IVF for tubal and male infertility in the Infertility Department of Shariati Hospital affiliated with Tehran University of Medical Sciences (TUMS). A history of PCOS, autoimmune diseases, menstrual disturbance, endometriosis, hirsutism, and hyperprolactinemia was regarded as exclusion criteria. 

Purification of GCs was done according to previous studies (8). First, to eliminate the individual’s effects, follicular fluids obtained from different participants were pooled and centrifuged at 3000 rpm for 10 minutes. The cell pellet was resuspended in Dulbecco’s Modified Eagle Medium F-12 (DMEM/F-12, Gibco, Finland), then layered over Ficoll-Paque (GE Healthcare Biosciences, Uppsala, Sweden) and centrifuged at 3000 rpm for 10 minutes. We collected GCs from the interphase, and washed, and cultured them in a complete medium that contained DMEM/F-12 supplemented with 10% (v/v) heat-inactivated fetal bovine serum (FBS, Gibco, South America), 100 mg/ml of streptomycin (Gibco by Life Technologies, Auckland, New Zealand), 100 U/ml of penicillin (Gibco by Life Technologies, Auckland, New Zealand), 2 mmol/l of glutamax (Sigma, St Louis, MO, USA), and 2 mg/ml of amphotericin B (PAN Biotech, Berlin, Germany) at 37˚C in a humidified atmosphere containing 5% CO_2_ , for different experiments. The medium was freshly changed every other day. In the present study, we defined the study groups as control, cells treated with dimethyl sulfoxide (DMSO, Sigma, Germany), H_2_ O_2_ , AST, AST+H_2_ O_2_ , and Trig+AST+H_2_ O_2_.

### Measurement of cell viability

To evaluate the viability of GCs after treatment with H_2_ O_2_ and AST (Sigma, China) and to determine an optimum dose for GCs treatment, 3-(4,5-dimethylthiazol-2-yl)-2,5-diphenyltetrazolium bromide (MTT, Alfa Aesar by Thermo Fisher Scientific, Germany) test was conducted according to previous studies (9, 10). It should be noted that AST was prepared by dissolving in DMSO. The test depends on the ability of viable cells to reduce tetrazolium bromide by mitochondrial dehydrogenase to produce formazan crystals (11). Briefly, GCs were cultured in a 96- well plate at a density of 1×10^4^ cells per well and treated with H_2_ O_2_ (Fluka, Germany) at concentrations of 100, 150, 200, 300, and 400 µM for 2 hours at 37˚C, to evaluate the cell viability after oxidative stress. Next, MTT solution at a concentration of 0.5 mg/ml was added to each well and plates were dark incubated at 37˚C for 4 hours. DMSO was used for dissolving the produced colorful crystals and the optical density (OD) of samples was estimated at 570 nm by a microplate reader (EONTM, BioTek, USA) with a background control as the blank. Moreover, GCs were treated with various concentrations of AST (0, 5, 10, and 20 µM) for 24 hours at 37˚C using MTT assay for evaluating the cell viability as described above. Finally, the MTT assay was conducted after pretreating cells with different concentrations of AST (0, 5, and 10 µM) for 24 hours followed by H_2_ O_2_ treatment at a concentration of 200 µM for another 2 hours at 37˚C to determine the optimal dose of AST for next steps.

### Measurement of reactive oxygen species levels

The intracellular ROS levels were calculated using 2’-7’-Dichlorodihydrofluorescein diacetate (DCFH-DA, Sigma, Switzerland) fluorescent probe by flow cytometry (12). Briefly, cells were cultured at a density of 2×10^5^ cells per well in the presence or absence of 5 µM of AST for 24 hours and then, exposed to 200 µM H_2_ O_2_ for another 2 hours at 37˚C. In addition, Trig (Sigma, Switzerland) at a concentration of 0.1 µM was used as an inhibitor of NRF2, 1 hour before treatment with AST. Then, GCs were incubated with 1 µM of DCFH-DA for 30 minutes at 37˚C and resuspended in phosphate buffered saline (PBS, Sigma, Germany). Flow cytometry was applied for detecting the fluorescence intensity at the wavelength of 525nm (FL1-H) band-pass filter based on mean fluorescence intensity of 10,000 cells. FlowJo 7.6.1 was applied for analyzing the results.

### Apoptosis assay

The Annexin V FITC-Propidium Iodide (PI) Apoptosis Detection Kit (Invitrogen by Thermo Fisher Scientific eBioscience) was applied to determine the total cell apoptosis according to the manufacturers’ protocol. In details, GCs were cultured in a six-well plate at a density of 1×10^5^ cells per well in the presence or absence of 5 µM of AST for 24 hours and then, treated with 200 µM H_2_ O_2_ for another 2 hours at 37˚C. Moreover, the effect of Trig at a concentration of 0.1 µM on GCs viability was measured. Next, GCs were resuspended in 1X Annexin-Binding buffer and a dark incubation at room temperature for 15 minutes was accomplished after adding Annexin V-FITC and PI. The fluorescence emission was measured by flow cytometry. Staining for apoptosis was performed as described by the manufacturer. Annexin V-negative, PI-negative stained cells: viable cells; Annexin V-positive, PI-negative stained cells: early apoptotic cells; Annexin V-positive, PI-positive stained cells: late apoptotic cells; and Annexin V-negative, PI-positive stained cells: necrotic cells (13). The stained cells were analyzed by FlowJo software. 

### Real-time polymerase chain reaction

Total RNA was extracted from cells using TRIzol reagent (Life Technologies, Gaithersburg, MD, USA) as explained by the manufacturer. Next, 1 μg of total RNA was applicable for cDNA synthesis using a First-Strand cDNA Synthesis Kit (Thermo scientific, Foster City, CA, USA) based on the manufacturer’s protocol. Real-time polymerase chain reaction (real-time PCR) was conducted to quantitate mRNA levels by the RealQ plus 5x Master Mix Green (Bio-Rad Laboratories, Hercules, CA, USA) using an Applied Biosystem StepOne real-time PCR, according to the manufacturers’ protocol. *GAPDH* was used as an internal standard for normalizing the expression levels of our studied genes using the 2^-ΔΔCt^ method in order to obtain the relative fold change results (14). All samples were analyzed in triplicate. The primers used in the present study are shown in Table 1.

**Table 1:** Forward and reverse primers used for real-time polymerase chain reaction


Primer	Primer sequence (5ˊ-3ˊ)

*NRF2*	F: TTCCTTCAGCAGCATCCTCTC
	R: AATCTGTGTTGACTGTGGCATC
*GCLC*	F: GGGCGATGAGGTGGAATAC
	R: GGGTAGGATGGTTTGGGTTTG
*GCLM*	F: GCGGTATTCGGTCATTGTG
	R: GGTAAGTTATGCTCCTAAGTCAG
*HO1*	F: TGACACCAAGGACCAGAGC
	R: TAAGGACCCATCGGAGAAGC
*NQO1*	F: TATCCTGCCGAGTCTGTTCTG
	R: AACTGGAATATCACAAGGTCTGC
*GAPDH*	F: AGTCCACTGGCGTCTTCAC
	R: ATCTTGAGGCTGTTGTCATACTTC


### Western blot analysis 

GCs were lysed by a Protein Extraction Kit (Active Motif Inc., Carlsbad, CA, USA) based on the manufacturer’s protocol. The insoluble material was removed by centrifugation at 15000×g for 10 minutes at 4˚C. After collecting supernatants, Bradford reagent (Bio-Rad, Foster City, MI, USA) was used to determine the protein concentrations. Protein lysates at a concentration of 20 µg/µl were subjected to sodium dodecyl sulfate-polyacrylamide gel electrophoresis (SDS-PAGE) and then, transferred to polyvinylidene difluoride membranes (Bio-Rad). After blocking with 5% BSA in TBST buffer at 4˚C overnight, the membranes were blotted with primary antibodies including antibody against NRF2 (1:750; GeneTex, USA), antibody against KEAP1 (1:750; Abcam, Cambridge, MA, USA), and antibody against β-actin (1:500; Santa Cruz Biotechnology, CA, USA), at 4˚C overnight. Next, the blots were washed and incubated with corresponding horseradish peroxidase (HRP)-linked secondary antibodies (rabbit anti-mouse IgG, ab97046, 1:5000; Abcam, Cambridge, UK) for 2 hours at room temperature. Protein bands were developed using a chemiluminescence system (ECL-plus, Lumigen, Inc., Southfield, MI, USA) (15). β-actin was used as an internal protein to normalize the expression of target proteins NRF2 and KEAP1. Data were analyzed by the ImageJ software.

### Measurement of NRF2 activity

We used a TransAM NRF2 Transcription Factor ELISA Kit (Active Motif Inc., Carlsbad, CA, USA) to evaluate the binding activity of NRF2 to DNA according to the manufacturer’s protocol. In detail, we incubated 2.5 μg of nuclear extracts in a 96-well plate after ARE oligonucleotides immobilization. By washing and adding an NRF2 antibody, we incubated the plate again and finally used a HRP-linked secondary antibody to provide colorimetric data. A microplate reader was applied for detecting the absorbance at 450 nm. A_450_ indicated the binding activity of NRF2-ARE.

### Statistical analysis

The Statistical Package for Social Sciences 22 (SPSS 22, Inc., Chicago, IL, USA) was used for the statistical analysis of all results. The Kolmogorov–Smirnov test was used for testing the normalization of data. For multiple comparisons between groups, Mann-Whitney U-test was used for nonparametric data. Results are shown as mean ± standard deviation. Values of P<0.05 were regarded as significant.

## Results

### The viability of granulosa cells after H_2_ O_2_ and Astaxanthin treatments

**Fig.1:**
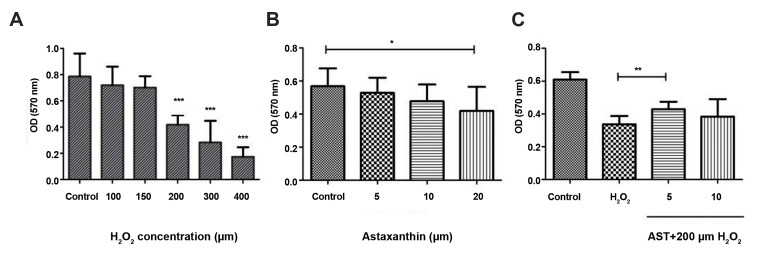
H_2_ O_2_ and AST toxicity measurement and the protective effect of AST against H_2_ O_2_ in GCs. **A. **To evaluate oxidative stress conditions; GCs were treated with various concentrations of H_2_ O_2_ (100, 150, 200, 300, and 400 µM) for 2 hours. **B. **To determine AST toxicity on GCs, various concentrations of AST (0, 5, 10, and 20 µM) were used for 24 hours. **C.** To evaluate the protective effects of AST on oxidative stress conditions, GCs were treated with various concentrations of AST (0, 5, and 10 µM) for 24 hours, next treated with 200 μM of H_2_ O_2_ for another 2 hours. Results are demonstrated as the mean ± SD. *; P<0.05, **; P<0.01, and ***; P<0.001, GCs; Granulosa cells, AST; Astaxanthin, and OD; Optimal density.

To determine the viability of GCs following treatment with various concentrations of AST (0, 5, 10, and 20 µM), we used MTT assay which showed a significant cell death at a concentration of 20 µM compared to the control group. Moreover, we also used MTT assay for determining the most appropriate concentration of H_2_ O_2_ for induction of oxidative stress. Thus, we treated GCs with different concentrations of H_2_ O_2_ (100, 150, 200, 300, and 400 µM) for 2 hours. We detected a reduction of cell viability up to 50% at 200 µM and higher concentrations after 2 hours (P<0.001). Finally, we pretreated our studied cells with various concentrations of AST (0, 5, and 10 µM) for 24 hours followed by an extra treatment with 200 µM H_2_ O_2_ for 2 hours, to find the optimal dose of AST for protecting GCs from oxidative damage as provided in Figure 1. Here, the optimal dose of AST with the best protective effect was 5 µM for 24 hours.

### Astaxanthin inhibits reactive oxygen species production

**Fig.2:**
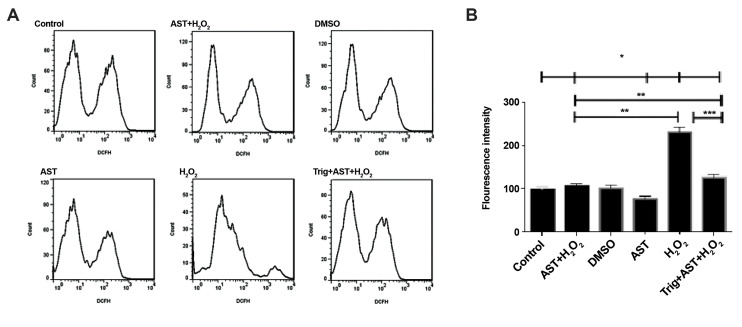
ROS induction. **A, B. **AST protects GCs from H_2_ O_2_ -mediated ROS generation. GCs were pretreated with 5 µM of AST for 24 hours, and then treated with 200 µM of H_2_ O_2_ for another 2 hours. GCs were also treated with 0.1 µM of Trig 1 hour before the exposure to AST. Intracellular ROS levels were evaluated by flow cytometry using a DCFH-DA fluorescent probe. Values are presented as the median fluorescence ± SD of 3 independent experiments. *; P<0.05, **; P<0.01, ***; P<0.001, ROS; Reactive oxygen species, AST; Astaxanthin, GCs; Granulosa cells, DMSO; Dimethyl sulfoxide, and Trig; Trigonelline.

Intracellular ROS levels were evaluated by a DCFH-DA fluorescent probe. For this purpose, we pretreated cells with 5 µM of AST for 24 hours and then, treated them with 200 µM of H_2_ O_2_ for another 2 hours. Here, we observed a significant increase in ROS generation using a DCF fluorescence, in the H_2 _O_2_ -treated group (mean fluorescence: 215 vs. 93) as shown in Figure 2, which was remarkably reduced to 50% after pretreatment with AST in the AST+H_2_ O2 and Trig+AST+H_2_ O_2_ groups (P<0.01 and P<0.001, respectively). The fluorescence intensity of GCs was significantly decreased after AST pretreatment in all AST-treated groups.

### Astaxanthin prevents granulosa cells from H_2_ O_2_ - induced apoptosis 

**Fig.3:**
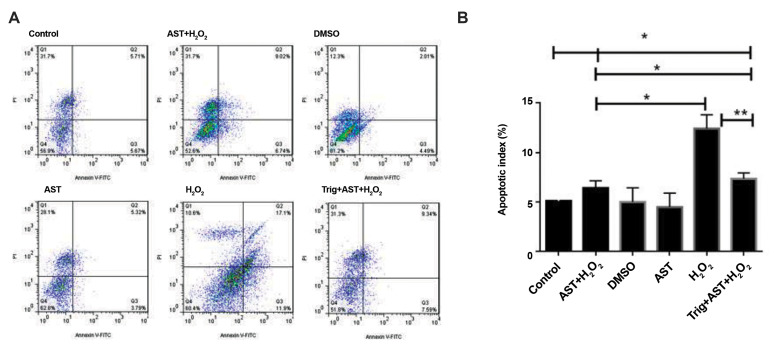
AST protects GCs from H_2_ O_2_ -induced apoptosis. **A. **The apoptosis rate was measured by an annexin V/PI double staining test and flow cytometry. The quadrants on annexin V/PI dot plots classed as: Annexin V-negative, PI-negative staining cells: viable cells; Annexin V-positive, PI-negative staining cells: early apoptotic cells; Annexin V-positive, PI-positive staining cells: late apoptotic cells; and Annexin V-negative, PI-positive staining cells: necrotic cells. **B. **The quantitative data are shown as median ± SD of three independent experiments. *; P<0.05, **; P<0.01, AST; Astaxanthin, GCs; Granulosa cells, DMSO; Dimethyl sulfoxide, and Trig; Trigonelline.

H_2_ O_2_ exposure as a common model used for the induction of oxidative damage, increases cellular apoptosis. To determine the protective effect of AST against H_2_ O_2_ -induced apoptosis, GCs were pretreated with 5 μM of AST as the optimal dose for 24 hours and then, treated with 200 µM of H_2_ O_2_ for 2 hours. The annexin V/PI staining was performed to determine the total GCs apoptosis due to H_2_ O_2_ treatment with or without pretreatment of AST by using flow cytometry. As provided in Figure 3, annexin V+/PI- as an indicator of early apoptotic cells percentage showed a higher apoptosis rate in the H_2 _O_2_ -treated GCs in comparison with the control group which was significantly reduced after pretreatment with AST (P<0.05). Moreover, GCs were treated with 0.1 µM of Trig as a known inhibitor of NRF2 1 hour before treatment with AST. Remarkably, the apoptosis rate was still significantly lower in the Trig+AST+H_2_ O_2_ GCs compared to H_2_ O_2_ -treated GCs (P<0.01) but Trig treatment slightly increased the percentage of early apoptotic cells compared to the AST+H_2_ O_2_ group (P<0.05).

### Astaxanthin enhances gene and protein expression and nuclear localization of NRF2 while declines KEAP1 protein levels

**Fig.4:**
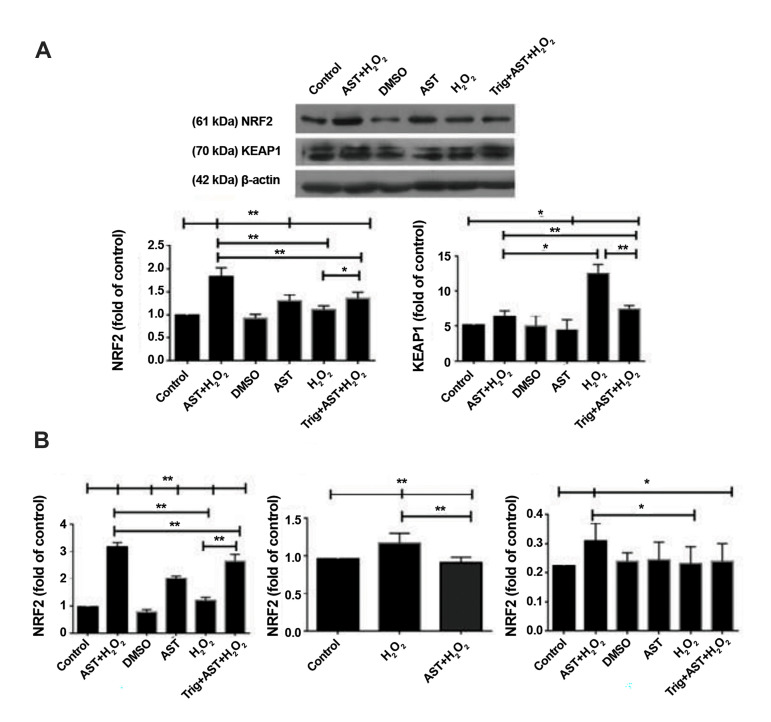
Evaluation of NRF2 mRNA, protein, and activity and KEAP1 protein levels after AST and H_2_ O_2_ treatment in GCs. **A.** NRF2 and KEAP1 protein levels were evaluated by western blot after treatment with 5 µM of AST for 24 hours and then treatment with 200 µM of H_2_ O_2_ for another 2 hours. The band densities of NRF2 and KEAP1 were normalized against β-actin. **B, C.** Real-time PCR was conducted to evaluate the expression of *NRF2* mRNA. *GAPDH* was used as an internal standard for normalization. **D.** Effects of AST on DNA binding activity of NRF2. The molecular weights of NRF2, KEAP1, and β-actin are reported to be 61, 70, and 42 kDa, respectively. The data are indicated as the mean ± SD of 3 independent experiments. *; P<0.05, **; P<0.01, AST; Astaxanthin, GCs; Granulosa cells, PCR; Polymerase chain reaction, DMSO; Dimethyl sulfoxide, and Trig; Trigonelline.

Firstly, our results indicated that H_2_ O_2_ treatment resulted in induced expression of NRF2. The protein levels of KEAP1 as an endogenous inhibitor of NRF2, was also increased after H_2_ O_2_ exposure, however, it was not significant. Moreover, pretreatment with AST significantly induced *NRF2* expression at both mRNA and protein levels in cells with or without H_2_ O_2_ exposure (P<0.01). Moreover, AST increased NRF2 activity and its connection to ARE region in DNA compared to the H_2_ O_2_ -treated GCs without AST exposure (P<0.05) which was induced by Trig treatment as an inhibitor of NRF2 (P<0.05). AST also reduced KEAP1 protein levels in cells with or without H_2_ O_2_ exposure (P<0.05). Furthermore, Trig resulted in a significant decrease in *NRF2* gene expression in GCs treated with H_2_ O_2_ (P<0.01, Fig.4A-C). It also significantly reduced gene and protein expression of *NRF2* compared to the H_2_ O_2_ -treated group after AST pretreatment (P<0.01). The protein levels of KEAP1 were significantly induced in the Trig+AST+H_2_ O_2_ group in comparison to the H_2_ O_2_ - treated group with or without AST pretreatment (P<0.01). However, as provided in Figure 4, the higher expression of *NRF2* at both mRNA and protein levels and the lower protein levels of KEAP1 were still observed as significant after Trig exposure in the Trig+AST+H_2_ O_2_ GCs compared to the H_2_ O_2_ -treated GCs.

### Astaxanthin increases the expression of *GCLC, GCLM, HO1*, and *NQO1* genes

**Fig.5:**
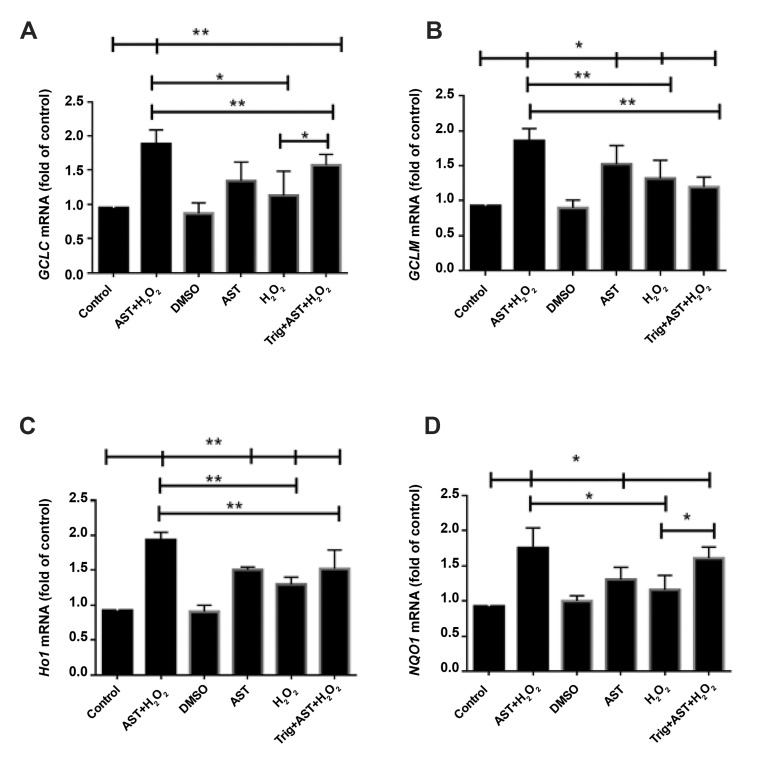
Evaluation of *GCLC, GCLM, Ho1, *and *NQO1 *mRNAs after AST and H_2_ O_2_ treatment in GCs. After treatment with 5 µM of AST for 24 hours and then treatment with 200 µM of H_2_ O_2_ for another 2 hours, **A.** *GCLC,*** B.** *GCLM, ***C. ***Ho1,* and **D. ***NQO1 *mRNAs were measured using real-time PCR. *GAPDH* was used as an internal standard for normalization. The data are indicated as the mean ± SD of 3 independent experiments. *; P<0.05, **; P<0.01, AST; Astaxanthin, GCs; Granulosa cells, PCR; Polymerase chain reaction, DMSO; Dimethyl sulfoxide, and Trig; Trigonelline.

To determine whether the effect of AST on the activation of NRF2/ARE pathway is followed by a higher gene expression of phase II enzymes including *GCLC, GCLM, HO1,* and *NQO1*, we conducted real-time PCR. The gene expression of these antioxidant enzymes was increased after exposure to H_2_ O_2_ in comparison with the control group. Our results demonstrated that pretreatment with 5 µM of AST for 24 hours, significantly enhanced the gene expression of phase II enzymes in the H_2_ O_2_ -treated and untreated groups (Fig.5). Moreover, Trig as an inhibitor of NRF2, significantly attenuated this effect on the mRNA levels of *GCLC, GCLM*, and *HO1* in the H_2_ O_2_ -treated GCs after pretreatment with AST (P<0.01). Therefore, these findings support the role of phase II antioxidant enzymes in the protective effects of AST through *NRF2* up-regulation. 

## Discussion

In the present study, we intended to examine the effects of AST on H_2_ O_2_ -induced oxidative stress in primary human GCs through investigating the expression of *NRF2, KEAP1* and downstream phase II enzymes including *GCL, HO1, *and *NQO1*. The main finding of our study was the stimulatory effect of AST on the gene and protein levels and the nuclear localization of NRF2 along with its inhibitory effect on KEAP1 protein levels. Importantly, we indicated that AST pretreatment suppressed ROS generation and cell death in GCs under the conditions of oxidative stress. Moreover, we revealed that using Trig as an inhibitor of *NRF2*, reduced the protective effects of AST by decreasing *NRF2* expression and activity and the gene expression of phase II enzymes. However, its inhibitory role did not completely remove the protective effects of AST on GCs. Therefore, our study may support a key role of the NRF2/ARE pathway in stimulating antioxidant enzymes induced by AST pretreatment in GCs. 

As mentioned before, oxidative stress plays an important role in GCs related disorders like PCOS and may have a notable influence on IVF outcome. Therefore, developing an accurate model of oxidative stress is essential in different studies. One of the most applicable models used for establishing oxidative stress is the treatment of cultured cells like primary human GCs with H_2 _O_2_ (4). Here, we used the model of H_2_ O_2_ -induced oxidative stress in GCs for investigating the antioxidant efficiency against oxidative damage. 200 µM of H_2_ O_2_ for 2 hours was determined to induce oxidative stress in GCs for the next experiments. In our recent study, we showed that a concentration of 200 μM of H_2_ O_2 _for 2 hours, promotes oxidative stress in GCs (16). A recent similar study also used 200, 400, and 600 μM of H_2_ O_2_ for 48 hours to induce oxidative stress in GCs and evaluate the expression of *NRF2* and associated antioxidant enzymes (17). In addition, another study used 200 µM of H_2_ O_2_ for 24 hours to create the same condition in retinal pigment epithelial cells (18). Furthermore, 400 µM of H_2_ O_2_ for 48 hours was used to trigger the model of oxidative stress in GCs (17). Our chosen concentration of H_2_ O_2_ for creating the model of oxidative stress was almost supported by other studies too in different cultured cells like human keratinocytes (19).

Antioxidant enzymes are produced more under conditions of excess ROS production to neutralize oxidative stress and return the homeostasis of cells like GCs and then, regulate ovarian follicles growth and function. Earlier studies emphasized the importance of NRF2 and KEAP1 in the regulation of GCs condition at several phases of follicles (17). Since the exact mechanisms underlying the interaction between oxidative stress and antioxidant defense in human GCs, are largely unknown and need more comprehensive investigations, here, we intended to explore the role of NRF2/ARE pathway in protecting GCs against ROS production and apoptosis by using the natural carotenoid pigment, AST.

Recently, AST has been under great attention for its various biological functions including ROS scavenging, anti-inflammatory, anti-apoptotic, and anti-oxidative effects (20). AST is a powerful carotenoid antioxidant having many potential applications in human health protection. Although the exact mechanism of AST in reducing oxidative damage is largely unknown, the role of NRF2/ARE pathway activation for these anti-oxidative effects was illustrated (21). However, the protective effect of AST on human GCs against oxidative stress is still elusive. Here, we firstly determined the best protective concentration of AST. The optimal dose of AST we used was 5 µM for 24 hours which was also supported by other researches done under similar concentrations. For instance, 5 µM of AST was able to protect keratinocytes and peritoneal mesothelial cells from oxidative damage (22, 23). Likewise, AST at concentrations of 5 and 6.25 μM increased the expression of phase II antioxidant enzymes in other types of cells (24).

One of the most important results of our study is that AST can inhibit ROS production and protect cells from apoptosis. The intracellular ROS levels generated by H_2_ O_2 _were significantly lower in GCs after pretreatment with 5 µM AST. Several studies demonstrated that AST acts as a potent free radical scavenger and reduces the amount of intracellular ROS in several types of human cells. For instance, AST at a concentration of 5 μM protected peritoneal mesothelial cells by scavenging glucose-induced ROS (23). Interestingly, another study showed that AST at concentrations of 10 and 20 μM decreased ROS production in retinal pigment epithelial cells (18). Moreover, 2 μM of AST decreased fatty acid-induced ROS production in human lymphocytes (25). Several studies also reported the preventive effects of AST on ROS production in human neuroblastoma cells (26). AST at concentrations of 10 and 100 μM also scavenged intracellular ROS in retinal ganglion cells (27). Putting these together, we may conclude that AST as a direct scavenger of free radicals like H_2_ O_2_ , has beneficial effects on improving viability of primary human GCs.

In our study, flow cytometry analysis showed a possible role of AST in decreasing H_2_ O_2_ -induced GCs early and late apoptosis through its anti-oxidative and anti-apoptotic properties. The present study suggests that AST up-regulated NRF2/ARE pathway and as a result, suppressed the apoptosis rate of GCs induced by intracellular ROS. Along with our data, other studies also suggested the inhibitory role of AST on apoptosis in different human cell types including keratinocytes treated with 5 µM AST (22), alveolar epithelial cells treated with 8 µM AST (28), and human neuroblastoma cells treated with 20 μM AST (29). Therefore, AST can be used for protecting GCs from apoptosis through its scavenging activity and then, to strengthen the ability to reproduce in women. Moreover, as high levels of NRF2 were observed in the presence of AST followed by a decrease in ROS production and cell damage, we may suggest NRF2 as a survival protein in follicular development.

Among many endogenous antioxidant components involved in maintaining cellular homeostasis, NRF2- ARE pathway and its underlying targets phase II enzymes GCL, HO1, and NQO1, are of great importance (2). They are induced under conditions of oxidative stress when NRF2 as a key transcription factor, is translocated into the nucleus for binding to ARE region leading to enhanced expression of phase II antioxidant enzymes. But until the homeostasis of the cell remains normal, NRF2 is inactivated by its endogenous inhibitor, KEAP1 protein (30). As described earlier, the phase II enzymes induced by this pathway consist of several antioxidants. Here, we intended to evaluate the effects of AST treatment on mRNA and protein expression and the activity of NRF2 as well as the gene expression of *GCLC, GCLM, HO1,* and *NQO1*. Furthermore, we evaluated the protein levels of KEAP1 to investigate whether the effects of AST on the NRF2-ARE pathway is dependent on NRF2 inhibitor or not. Our study demonstrated that AST pretreatment induced the gene and protein levels of *NRF2* but reduced the protein levels of KEAP1 in GCs in the presence or absence of H_2_ O_2_ . Moreover, measurement of NRF2 activity showed that AST was able to significantly increase NRF2 translocation to the nucleus and its connection to the ARE consensus site (5′-GTCACAGTGACTCAGCAGAATCTG-3′) compared to H_2_ O_2_ -treated GCs without AST. Then, AST stimulates NRF2-ARE pathway by both enhancing the gene expression and activity of NRF2 and decreasing KEAP1 protein levels. Interestingly, a higher level of NRF2 in the nucleus may result from its up-regulation and lower levels of KEAP1 protein as its intracellular inhibitor. 

We also observed a significant increase in the gene expression of phase II enzymes after AST pretreatment in H_2_ O_2_ -treated and untreated groups which followed by subsequent protection of GCs against oxidative damage and cell death. Some studies support our findings including a recent study that indicated the potential role of AST in increasing the nuclear localization of NRF2 and subsequent expression of *NQO1* and *HO1* in glomerular mesangial cells (31). Interestingly, AST induced the gene expression of *HO1* followed by the activation of NRF2 nuclear translocation in human umbilical vein endothelial cells which was reduced after using NRF2 specific small interfering RNA (siRNA) for its inhibition (32). Furthermore, AST reduced the levels of KEAP1 protein which triggered its dissociation from NRF2 and increased the nuclear localization of NRF2 in the kidney of diabetic rats (33). Another investigation on the brain after experimental subarachnoid hemorrhage showed that AST can activate NRF2-ARE pathway and subsequent gene expression of *HO1* and *NQO1* enzymes, then ameliorated oxidative stress (34). Moreover, AST at a concentration of 6.25 μM produced the highest gene expression of *NRF2, NQO1*, and *HO1* compared to other concentrations of AST in HepG2 cells (24). In another similar study on retinal pigment epithelial cells, researchers reported a higher NRF2 nuclear localization and GCLC, GCLM, HO1, and NQO1 expression after treatment with 5, 10, and 20 μM of AST (18). In view of our findings and the others, we may remark the importance of NRF2-ARE pathway in the activation of its downstream phase II antioxidant enzymes for the protection of ovarian follicles, preventing women from oxidative stress-related disorders, and increasing the success rates of IVF. Here, we pointed towards the activation of NRF2-ARE pathway by AST as well as other antioxidants such as phenolic compounds and carotenoids (35). Finally, we hope to achieve a better pregnancy result by applying AST as an inducer of NRF2/ARE pathway to neutralize oxidative stress and apoptosis in human GCs.

Moreover, Trig as an alkaloid derived from niacin (vitamin B3) was added at a concentration of 0.1 µM to express the importance of NRF2 in the protective effects of AST and describe the link between NRF2-ARE pathway and AST-induced phase II enzymes expression (7). Here, we observed a significant decrease in the expression of *NRF2* at both mRNA and protein levels as well as the gene expression of phase II enzymes by adding Trig to H_2_ O_2 _-treated and untreated GCs after AST pretreatment. Likewise, Trig treatment induced the levels of endogenous inhibitor of NRF2, KEAP1 protein along with a reduction in NRF2 activity in H_2_ O_2_ -treated GCs after AST pretreatment. This underlines the remarkable role of AST-induced NRF2/ARE pathway in stimulating phase II enzymes. However, our results revealed that the protective effects of AST on our studied target expression remained significant after Trig treatment compared to H_2_ O_2_ -treated GCs which highlighted the effectiveness of AST and the inability of Trig to completely erase the protective effects of AST. Furthermore, there are other studies regarding the inhibitory role of Trig on the nuclear accumulation of NRF2 protein in different types of cells (36). According to our data and those reported by previous studies, it seems likely that Trig inhibits NRF2 pathway and its downstream antioxidant enzymes mostly by inhibition of NRF2 nuclear accumulation. Putting these findings together, our study established the importance of NRF2/ARE pathway in the related antioxidant defense induced by AST regarding the possibility that Trig has an inverse influence on the stimulatory role of AST on GCLC, GCLM, HO1, and NQO1 expression.

Altogether, in this study, we showed that AST as a protective natural factor promotes gene and protein levels of NRF2 and inhibits the protein levels of KEAP1 in primary human GCs. We may consider this mechanism for the inhibition of H_2_ O_2_ -induced apoptosis and intracellular ROS generation by AST treatment. Hence, here for the first time, we showed that AST inhibits H_2_ O_2_ -induced apoptosis and intracellular ROS generation through a mechanism by which NRF2 induces the expression of antioxidant enzymes such as GCL, HO1, and NQO1 in GCs. Therefore, the current study provides supporting data considering the possible role of AST in presenting a noble therapeutic strategy for infertility, PCOS and other ovarian diseases related to oxidative damage. These results show that AST as a radical scavenger and an anti-apoptotic factor, probably protects primary human GCs against H_2_ O_2_ -induced oxidative stress and cell death via regulating NRF2 and related factors and thus, improves the development of the ovarian follicles.

The limitations of the present study included using Trig as an inhibitor of *NRF2*. Because this agent is not capable of completely suppressing *NRF2* as provided in our results, a more applicable and specific material must be applied for complete inhibition of *NRF2* to investigate its role in activating downstream antioxidant defense. Therefore, we suggest the use of a specific siRNA for this propose in future studies related to this topic. 

## Conclusion

Our study demonstrated that AST promotes gene and protein levels of NRF2 and inhibits the protein levels of KEAP1 in primary human GCs. It seems likely that activation of NRF2 by AST may attenuate oxidative stress in human GCs through activation of downstream antioxidant enzymes including GCL, HO1, and NQO1 and may produce better outcomes of IVF and reproduction in women.
